# “Inflammaging” and bone in the OsteoLaus cohort

**DOI:** 10.1186/s12979-020-00177-x

**Published:** 2020-03-05

**Authors:** Jessica Fischer, Didier Hans, Olivier Lamy, Pedro Marques-Vidal, Peter Vollenweider, Bérengère Aubry-Rozier

**Affiliations:** 1 Faculty of Biology and Medicine, Lausanne University, Unicentre, 1015 Lausanne, Switzerland; 2 Center for Bone Diseases, Lausanne University Hospital and University of Lausanne, 1011 Lausanne, Switzerland; 3 Department of Internal Medicine, Lausanne University Hospital and University of Lausanne, 1011 Lausanne, Switzerland; 4 Division of Rheumatology, Lausanne University Hospital and University of Lausanne, 1011 Lausanne, Switzerland

**Keywords:** Osteoporosis, Inflammation, Bone mineral density, Trabecular bone score, Cytokines

## Abstract

**Background:**

“Inflammaging” is a coined term that combines the processes of inflammation (within the normal range) and aging, since chronic, low-grade, systemic inflammation emerges with increasing age. Unlike high-level inflammation, with which deleterious effects on bone no longer need to be demonstrated, it is unclear whether inflammaging exerts deleterious effects on bone too.

**Method:**

We assessed associations between inflammaging — measured via cytokine levels (high-sensitivity C-reactive protein (hs-CRP); interleukin-*1β* (IL-*1β);* interleukin-6 (IL-6) and tumor necrosis factor-α (TNF-α)) — and bone parameters (prevalent and incident fractures, bone mineral density (BMD) and trabecular bone score (TBS)) in 1390 postmenopausal women from the OsteoLaus study.

**Results:**

Mean (±SD) age was 64.5 ± 7.6 and mean bone mass index (BMI) 25.9 ± 4.5 kg/m2. Median hs-CRP, IL-*1β,* IL-6 and TNF-α were 1.4 pg/ml, 0.57 pg/ml, 2.36 pg/ml and 4.82 pg/ml, respectively. In total, 10.50% of the participants had a prevalent, low-impact fracture; and, after 5-years of follow up, 5.91% had an incident, low-impact fracture. Mean T-score BMD was − 1.09 ± 1.53 for the spine, − 1.08 ± 1.02 for the femoral neck, and − 0.72 ± 0.96 for the total hip. Mean spine TBS was 1.320 ± 0.10. We found a positive association between hs-CRP and BMD at all sites, and between hs-CRP and the TBS, but none of these associations were significant after adjustment. We found no association between prevalent or incident fractures and hs-CRP. No association was found between IL-1β, IL6 and TNF-α and BMD, TBS or fractures.

**Conclusion:**

Our results suggest that bone imaging and structure parameters are not associated with the low-grade cytokine levels (within the normal range) observed with inflammaging.

## Summary

In this large population-based cohort, we did not find a relation between the coined concept of “inflammaging” (hs-CRP, IL-6, IL-1β and TNF-α within the normal range) and bone parameters, measured in terms of prevalent and incident fractures, bone mass density and trabecular bone score.

## Background

The “network theory of aging” argues that a concomitant progressive increase in proinflammatory status is a major characteristic of the aging process. This phenomenon is also referred to as “inflammaging”, which is a coined term merging “inflammation” and “aging”. The term was first proposed by Francheschi in 2000 to describe the low-grade (inflammatory cytokines within the normal range), chronic, systemic inflammation associated with the progressive stimulation/depletion of the immune system that occurs with aging, in the absence of overt infection [1–3]. In addition to inflammatory cytokines — like tumor necrosis factor-alpha (TNF-α) [4], interleukin-6 (IL-6) and interleukin-1 (IL-1) — being shown to increase with age, so have serum levels of inflammatory parameters like C-reactive protein (CRP) [5], with some parameters like CRP and IL-6 increasing as early as the third or fourth decade of life [6].

Several studies have linked inflammaging to pathologies like atherosclerosis, type 2 diabetes mellitus, vascular disease, Parkinson’s disease, dementia, Alzheimer’s disease and chronic obstructive pulmonary disease (COPD) [4, 6–9]. Osteoporosis (OP) and fragility fractures are more prevalent in inflammatory diseases, particularly rheumatoid arthritis (RA), systemic lupus erythematosus (SLE) and COPD, when compared to the healthy population [10]. These findings suggest that chronic low elevations of circulating cytokines contribute to the development of OP [4, 6–9]. The immune system plays an important role in bone remodeling, and proinflammatory cytokines (IL-6, TNF-α and IL-1) stimulate bone resorption in vitro [10, 11]. Experimental studies in animals suggest that some inflammatory cytokines — including IL-1, IL-6 and TNF-α — play an important role in the pathogenesis of OP [12].

Osteoporosis, a major health concern in all developed countries [13, 14], is characterized by low bone mineral density (BMD) and microarchitectural deterioration (as measured by the trabecular bone score, TBS [13, 15]), resulting in increased bone fragility. Eighty percent of individuals with OP are women, largely due to the marked loss in BMD that begins at menopause, secondary to the marked decrease in estrogen related to the loss of ovarian function [16]. Moreover, estrogen plays a key role in regulating the production and activity of inflammatory cytokines like IL-1, IL-6 and TNF-α [17]. In recent years, several studies have been conducted to establish a link between proinflammatory cytokines and OP, but their results are conflicting: some found a positive association between inflammatory cytokines and OP [17–19] while in other studies these associations have been inconsistent [20–22].

Since 2010, we have been conducting a prospective study, called OsteoLaus, to determine bone parameters: fractures, BMD and TBS in postmenopausal women living in Lausanne, Switzerland, and identify the determinants of bone loss and bone fragility [23]. The primary aim of the present study was to explore in a population without clinically meaningful inflammation disease the effect of hs-CRP, and secondary aim three others inflammatory parameters —, IL-6, IL-1β and TNF-α — on bone parameters: evaluated in terms of prevalent and incident low-impact fractures, BMD and TBS in the OsteoLaus population. In others words, if in a normal population with normal cytokines levels, the inflammaging was related to bone health degradation.

## Materials and methods

### Recruitment

The OsteoLaus cohort is a sub-study of the CoLaus/PsyCoLaus study, an ongoing prospective study aiming to assess the determinants of cardiovascular diseases using a population-based sample drawn from the city of Lausanne, Switzerland. The methods of both studies have been published previously [23–25]. The aim of OsteoLaus is to cross-sectionally and prospectively (follow-up every 2.5 years) compare different models of fracture risk prediction, and to see how information on micro-architecture could either improve or explain the differences between these models. Between September 2009 and September 2012, all women between 50 and 80 years old at the first CoLaus/PsyCoLaus follow-up visit were invited to participate 6 months later in OsteoLaus. Of the initial 1704 participants invited, 1500 (88%) accepted. Of these, 1475 women were included in the OsteoLaus baseline visit, while 1234 participated 5 years later in the second OsteoLaus follow-up visit. For this study, we used cytokines measured during the first follow-up CoLaus/PsyCoLaus study, bone data from the baseline OsteoLaus study, and incident fractures listed at the second OsteoLaus follow-up visit.

### Lifestyle, clinical and radiological data, fractures

At baseline, each patient completed a questionnaire about their potential clinical risk factors for fracture/osteoporosis, according to the Swiss Fracture Risk Assessment Tool -FRAX® assessment-, including conditions with potential effects on bone metabolism and immunosuppressant treatments like disease-modifying antirheumatic drugs (DMARDS) or other biological therapies. Patients had one spine (L1 to L4) and one femoral measurement using the Discovery A System (Hologic, Waltham, MA, USA), blinded central processing of TBS (TBS iNsight v3.0, Medimaps, Merignac, France) based on a previously-acquired AP spine DXA scan, and a vertebral fracture (VFA) assessment using the semi-quantitative approach of HK Genant [26].

Based on the literature, we consider a TBS of more than 1.31 normal; a TBS between 1.23 and 1.31 indicative of partially-degraded bone architecture; and a TBS less than 1.23 indicative of degraded architecture [15, 27, 28].

We considered only major osteoporotic fractures, including at least one fracture of a vertebra (clinical or radiologic from grade 2/3 on a vertebral fracture assessment), hip, pelvis, humerus or radius, either occurring spontaneously or after a fall from ≤ standing height.

### Cytokine measurements

Venous blood samples (50 ml) were drawn in a fasting state. Serum was preferred to plasma, as it has been shown that different anticoagulants may affect absolute cytokine levels differently. hs-CRP was assessed by immunoassay and latex HS (IMMULITE 1000-High, Diagnostic Products Corporation, LA, CA, USA) with maximum intra- and inter-batch coefficients of variation of 1.3 and 4.6%, respectively. Serum samples were kept at − 80 °C before assessing IL-1β, IL-6 and TNF-α and sent in dry ice to the laboratory. Cytokine levels were measured using a multiplexed particle-based flow cytometric cytokine assay, a methodology used in other studies [29]. Milliplex kits were purchased from Millipore (Zug, Switzerland). The procedures closely followed the manufacturer’s instructions. The analysis was conducted using a conventional flow cytometer (FC500 MPL, Beckman Coulter, Nyon, Switzerland). Lower detection limits for IL-1β, IL-6 and TNF-α were 0.2 pg/ml. Good agreement between signal and cytokine was found within the assay range (R^2^ ≥ 0.99). Intra and inter-assay coefficients of variation were 15 and 16.7% for IL-1β, 16.9 and 16.1% for IL-6, and 12.5 and 13.5% for TNF-α, respectively.

### Inclusion and exclusion criteria

All participants with at least one BMD and TBS measurement, one questionnaire and one cytokine measurement were eligible for inclusion. To maintain inflammatory levels close to the general population, major exclusion criteria were the following: any disease known to affect bone metabolism (neoplasia, inflammatory bowel disease, RA, SLE, ankylosing spondylitis and psoriatic arthritis), drugs taken by subjects known to have an effect (negative or positive) on bone metabolism, and a bone mass index (BMI) either less than 16 or above 40.

### Statistical analysis

Statistical analysis was conducted using Stata version 14.2 (StataCorp, College Station, TX, USA). We divided cytokine results into quartiles. Univariable and multivariable analyses were used to examine the associations between the inflammatory and bone parameters: fractures, BMD and TBS. For bivariable analysis, we used chi-square or Fisher’s exact test for categorical variables and analysis of variance (ANOVA) for continuous variables. For multivariable analysis, we used logistic regression for categorical variables and analysis of variance (ANOVA) for continuous variables. Multivariable models were adjusted for age (continuous), body mass index (continuous), diabetes (yes/no), smoking (never/former/current), alcohol consumption (none/1–20/21+ units/week), current calcium treatment (yes/no) and current hormone replacement therapy (HRT) status (yes/no). Statistical significance was considered for a two-sided test with *p* < 0.05.

## Results

### Clinical characteristics of participants

Of the 1475 initial participants, 77 (5.2%) were excluded: 49 (3.3%) due to a corticoid, immuno-suppressant treatment or chronic inflammatory disease, and 28 (1.9%) because of missing data for at least one of the inflammatory parameter. In addition, 8 (0.5%) were excluded because of a BMI less than 16 or above 40. The clinical characteristics of included and excluded participants are summarized in Table 1. No significant differences were found between groups for any variable studied, except for calcium and vitamin D supplements, HRT, and other osteoporosis treatments.

**Table 1:** Comparison between excluded and included participants

	Included (*N* = 1390)	Excluded (*N* = 77)	*P*-value
Age (years)	64.5 ± 7.6	63.7 ± 7.8	0.379
Body mass index (kg/m^2^)	25.9 ± 4.5	26.3 ± 4.7	0.440
Smoking (%)			0.425 §
Never	650 (46.8)	25 (41.7)	
Former	503 (36.2)	21 (35)	
Current	237 (17.1)	14 (23.3)	
Alcohol consumption (%)			0.381 §
None	409 (29.4)	27 (35.1)	
1–20 units/week	956 (68.8)	50 (64.9)	
21+ units /week	25 (1.8)	0 (0)	
Diabetes (%)	67 (4.8)	6 (8.2)	0.797
Osteoporosis (any location)	**139 (10.0)**	**10 (13.0)**	**0.713**
Calcium and vitamin D (%)	497 (35)	45 (58)	< 0.001
Hormone replacement therapy (%)	323 (23)	9 (11)	0.018
Other current OP treatment (%)	58 (4.2)	12 (15.5)	< 0.001 §

OP, osteoporosis. Results are expressed as number of participants (column %) or as mean ± standard deviation. Statistical analysis performed using chi-square or Fisher’s exact test (§) for categorical variables and student’s t-test for continuous variables

Among the 1390 included participants, mean (±SD) age was 64.5 ± 7.6, and mean BMI was 25.9 ± 4.5 kg/m2; 10.50% had a prevalent low-impact fracture. Mean BMD was 0.925 ± 0.163 g/cm^2^ for the spine (T score − 1.09 ± 1.53), 0.728 ± 0.114 g/cm^2^ for the femoral neck (T score − 1.08 ± 1.02) and 0.854 ± 0.118 g/cm^2^ for the total hip (T score − 0.72 ± 0.96); mean spine TBS was 1.320 ± 0.10. During the second follow-up visit, 5.91% of the analyzed participants had a non-traumatic or low-impact fracture.

### Association between bone imaging and structure parameters and cytokine levels

We found a positive association between the BMD at all sites, the TBS, and hs-CRP in univariable analyses (Table 2). However, none of these associations remained significant after adjusting for age, smoking, BMI, diabetes, alcohol consumption, HRT, calcium and vitamin D or other osteoporosis treatments. We identified no association between fractures and levels of hs-CRP (Table 2).

**Table 2:** Bone parameters (fracture, BMD and TBS) according to quartiles of hs-CRP

	First	Second	Third	Fourth	*P*-value^a^	*P*-value trend test^b^	*P*-value adjusted^c^
N	349	347	352	335			
hs-CRP, median [IQR]	0.5 [0.3–0.6]	1.0 [0.9–1.3]	2.0 [1.7–2.3]	4.8 [3.6–7.2]			
Traumatic fracture (%)	89 (25.5)	79 (22.7)	108 (30.6)	82 (24.4)	0.096		0.973
Major osteoporotic fracture (%)	31 (8.8)	43 (12.4)	41 (11.6)	31 (9.2)	0.345		0.748
Incident 5 years fractures	15/339 (4.4)	24/339 (7.1)	23/343 (5.2)	17/325 (5.2)	0.415		0.147
Spine average BMD	0.901 ± 0.154	0.916 ± 0.166	0.928 ± 0.158	0.957 ± 0.169	0.001	< 0.001	0.625
Spine average T-score	−1.32 ± 1.45	− 1.17 ± 1.56	− 1.1 ± 1.49	− 0.78 ± 1.59	< 0.001	< 0.001	0.717
Spine average TBS	1.304 ± 0.100	1.318 ± 0.102	1.326 ± 0.098	1.336 ± 0.107	0.0006	< 0.001	0.231
Femoral neck BMD	0.717 ± 0.116	0.718 ± 0.113	0.729 ± 0.104	0.752 ± 0.120	0.0002	< 0.001	0.781
Femoral neck T-score	−1.18 ± 1.04	−1.17 ± 1.02	−1.08 ± 0.94	−0.87 ± 0.18	0.0002	< 0.001	0.809
Femur total BMD	0.835 ± 0.114	0.845 ± 0.116	0.858 ± 0.115	0.879 ± 0.125	< 0.001	< 0.001	0.895
Femur total T-score	−0.88 ± 0.93	−0.79 ± 0.94	− 0.69 ± 0.93	−0.51 ± 1.02	< 0.001	< 0.001	0.823

Results are expressed as number of participants (%) or as mean ± standard deviation. Statistical analysis performed using chi-square for categorical variables and ANOVA for continuous variables^a^, p of trend^b^ and after multiple adjustment^c^ (age, BMI, smoker, diabetes, alcohol, calcium, HRT)

We found no association between IL-1β, IL6 or TNF-α and bone parameters (fractures, BMD or TBS) either before or after adjustment (Tables 3, 4, 5).

**Table 3:** Bone parameters (fracture, BMD and TBS) according to quartiles of interleukin 1β

	First	Second	Third	Fourth	*P*-value^a^	*P*-value trend test^b^	*P*-value adjusted^c^
N	381	279	329	329			
Interleukin 1β, median [IQR]	undetectables	0.32 [0.25–0.43]	1.13 [0.84–1.50]	5.86 [3.02–12.8]			
Traumatic fracture (%)	98 (25.7)	87 (31.1)	85 (25.8)	75 (22.8)	0.131		0.275
Major osteoporotic fracture (%)	54 (14.1)	27 (9.68)	30 (9.1)	31 (9.4)	0.090		0.176
Incident fracture 5 years	25/374 (6.6)	18/272 (6.6)	12/318 (3.7)	20/320 (6.2)	0.341		0.368
Spine average BMD	0.918 ± 0.163	0.937 ± 0.175	0.940 ± 0.157	0.910 ± 0.161	0.057	0.626	0.606
Spine average T-score	−1.16 ± 1.55	−1.00 ± 1.62	−0.94 ± 1.47	− 1.21 ± 1.52	0.07	0.773	0.801
Spine average TBS	1.318 ± 0.108	1.323 ± 0.108	1.329 ± 0.098	1.316 ± 0.095	0.339	0.941	0.329
Femoral neck BMD	0.724 ± 0.110	0.740 ± 0.125	0.732 ± 0.109	0.725 ± 0.115	0.257	0.903	0.683
Femoral neck T-score	−1.12 ± 0.99	−0.98 ± 1.12	−1.05 ± 0.98	− 1.11 ± 1.03	0.289	0.947	0.721
Femur total BMD	0.848 ± 0.118	0.860 ± 0.126	0.862 ± 0.116	0.851 ± 0.112	0.333	0.664	0.918
Femur total T-score	−0.77 ± 0.96	− 0.67 ± 1.03	−0.65 ± 0.94	− 0.74 ± 0.91	0.342	0.735	0.998

Results are expressed as number of participants (%) or as mean ± standard deviation. Statistical analysis performed using chi-square for categorical variables and ANOVA for continuous variables^a^, p of trend^b^ and after multiple adjustment^c^ (age, BMI, smoker, diabetes, alcohol, calcium, HRT)

**Table 4:** Bone parameters (fracture, BMD and TBS) according to quartiles of interleukin 6

	First	Second	Third	Fourth	*P*-value^a^	*P*-value trend test^b^	*P*-value adjusted^c^
N	333	330	326	329			
Interleukin 6, median [IQR]	0.41 [0.22–0.63]	1.52 [1.19–1.87]	3.84 [2.98–5.30]	22.01 [12.33–61.31]			
Traumatic fracture (%)	76 (22.8)	99 (30.0)	80 (24.5)	91 (27.6)	0.155		0.497
Major osteoporotic fracture (%)	32 (9.61)	39 (11.82)	36 (11.04)	35 (10.64)	0.832		0.997
Incident fracture 5 years	20/323 (6.2)	12/320 (3.7)	21/318 (6.6)	22/323 (6.8)	0.320		0.461
Spine average BMD	0.921 ± 0.168	0.932 ± 0.164	0.933 ± 0.155	0.915 ± 0.168	0.437	0.696	0.375
Spine average T-score	−1.13 ± 1.59	−1.03 ± 1.54	−1.01 ± 1.46	−1.18 ± 1.58	0.424	0.707	0.367
Spine average TBS	1.322 ± 0.104	1.326 ± .0105	1.319 ± 0.103	1.319 ± 0.098	0.773	0.509	0.525
Femoral neck BMD	0.724 ± 0.113	0.727 ± 0.114	0.741 ± 0.112	0.727 ± 0.199	0.234	0.424	0.391
Femoral neck T-score	−1.13 ± 1.01	−1.096 ± 1.02	−0.97 ± 1.00	−1.10 ± 1.07	0.223	0.397	0.361
Femur total BMD	0.848 ± 0.118	0.857 ± 0.121	0.869 ± 0.119	0.845 ± 0.112	0.046	0.945	0.979
Femur total T-score	−0.77 ± 0.96	−0.69 ± 0.98	− 0.59 ± 0.97	−0.79 ± 0.92	0.043	0.913	0.987

Results are expressed as number of participants (%) or as mean ± standard deviation. Statistical analysis performed using chi-square for categorical variables and ANOVA for continuous variables^a^, p of trend^b^ and after multiple adjustment^c^ (age, BMI, smoker, diabetes, alcohol, calcium, HRT)

**Table 5:** Bone parameters (fracture, BMD and TBS) according to quartiles of tumour necrosis factor α

	First	Second	Third	Fourth	*P*-value^a^	*P*-value trend test^b^	*P*-value adjusted^c^
N	330	331	329	329			
TNF-α, median [IQR]	1.74 [1.08–2.19]	3.71 [3.19–4.29]	6.18 [5.40–6.99]	12.23 [9.77–20.17]			
Traumatic fracture (%)	84 (25.4)	95 (28.7)	86 (26.1)	81 (24.6)	0.662		0.500
Major osteoporotic fracture (%)	28 (8.4)	40 (12.0)	36 (10.9)	38 (11.5)	0.456		0.436
Incident fracture 5 years	23/325 (7.1)	13/320 (4.1)	13/320 (4.1)	26/320 (8.1)	0.054		0.678
Spine average BMD	0.930 ± 0.168	0.920 ± 0.156	0.920 ± 0.164	0.931 ± 0.168	0.730	0.893	0.315
Spine average T-score	−1.05 ± 1.58	−1.13 ± 1.47	−1.14 ± 1.55	−1.02 ± 1.57	0.697	0.814	0.342
Spine average TBS	1.326 ± 0.102	1.318 ± 0.099	1.316 ± 0.108	1.326 ± 0.101	0.451	0.981	0.816
Femoral neck BMD	0.738 ± 0.118	0.718 ± 0.114	0.729 ± 0.115	0.734 ± 0.111	0.152	0.937	0.755
Femoral neck T-score	−1.00 ± 1.06	−1.17 ± 1.02	−1.08 ± 1.03	− 1.04 ± 1.00	0.176	0.942	0.759
Femur total BMD	0.864 ± 0.121	0.842 ± 0.113	0.857 ± 0.117	0.856 ± 0.119	0.108	0.817	0.557
Femur total T-score	−0.64 ± 0.99	−0.82 ± 0.91	−0.69 ± 0.96	−0.70 ± 0.97	0.109	0.806	0.543

Results are expressed as number of participants (%) or as mean ± standard deviation. Statistical analysis performed using chi-square for categorical variables and ANOVA for continuous variables^a^, p of trend^b^ and after multiple adjustment^c^ (age, BMI, smoker, diabetes, alcohol, calcium, HRT)

## Discussion

In our population without clinically meaningful inflammation disease, a cohort of 1390 postmenopausal women, we found no association between cytokine levels and bone parameters assessed by BMD, TBS or prevalent or 5-year incident fragility fractures.

### Relationship between bone imaging and structure parameters and hs-CRP

Our study showed no difference in fractures, BMD or TBS across the different quartiles of hs-CRP. CRP is the most studied inflammatory marker. Several studies have shown that hs-CRP is associated with lower BMD and/or an increased risk of fracture [12, 17, 18, 20, 21, 28, 30, 31], but others have failed to detect significant associations [20, 22, 30, 32, 33]. In the Geelong Osteoporosis study [30], fracture risk increased by 24–32% for each SD increase in CRP independently of BMD, prevalent vertebral fractures and bone turnover parameters. In the Rotterdam study, Oei et al. [32] showed that hs-CRP was associated with incident fracture risk when men and women were analyzed together. However, this association just reached statistical significance for females (HR = 1.05, 95 IC 1.00–1.11, *p* = 0.05), and was more significant in the oldest age category (> 73 years) and for a BMI between 24.5 and 27.5 kg/m2. Our participants were younger. Cauley et al. [19] found a 37% increased risk of fracture in the highest tertile of CRP (> 3.14 mg/l); but, after adjustment, this association did not retain significance (HR = 1.34, 95% IC 0.99–1.82). Therefore, the population was over 70 years old, 48.5% male and 42% black. Finally, in studies in which a positive association was detected, no information on patients with inflammatory disease was given and chronic inflammatory diseases could be confounding factors.

Regarding the lumbar spine BMD, discordant results could be explained by the presence of degenerative disease, very common in people older than 60 to 65 years [34]. Spinal osteoarthritis (OA) is known to increase BMD in the lumbar spine [34–36]; and it has been reported that individuals with OA have significantly higher hs-CRP levels [20]. Therefore, it is possible that OA changes associated with aging may have confounded the association between hs-CRP levels and spine BMD. However, we cannot explain the absence of any correlation between TBS and hs-CRP by degenerative diseases, since Padlina et al. [34] showed that TBS is not affected by degenerative diseases like OA. Moreover, we cannot compare our results to those of other studies, in the absence of published data between hs-CRP and bone microarchitecture in females. Rolland et al. suggested an association between higher hs-CRP and poor trabecular, but not cortical microarchitecture in the distal radius, as assessed by peripheral quantitative computed tomography (pQCT) in men age 72 and older, but not in younger men [37]. Watanabe et al. demonstrated that TBS was associated with hs-CRP levels in 61 Japanese male COPD patients [38], but there are no published data on cytokines in patients without a chronic inflammatory disease and TBS.

### Relationship between bone imaging and structure parameters and IL-1β

Our study identified no difference in fractures, BMD or TBS, in the different quartiles of IL-1β. Interleukin 1 is a prototypic proinflammatory cytokine that regulates a wide variety of cellular and tissue functions. There are two forms of IL-1: IL-1α and IL-1β. Several data suggest the involvement of IL-1 in bone destruction under pathological conditions, including RA and OP. Iwakura et al. [39] demonstrated that IL-1 also plays an important role in physiological bone metabolism in mice. In particular, IL-1 directly activates RANK (receptor activator of nuclear factor--*k*B) inducing RANKL ligand and promoting osteoclastogenesis. However, Bab et al. found conflicting results when examining the role of IL-1 in vivo [40].

Kim et al. [41] found no significant differences in BMD (lumbar spine or proximal femur) or in serum bone parameters levels across the IL-1α or IL-1β genotypes. Langdahl et al. [42] found that an 86-base pair repeat polymorphism in the IL-1 receptor antagonist (IL-1ra) gene was associated with an increased risk of osteoporotic fractures. Other polymorphisms in the IL-1ra and IL-1β genes were not associated with osteoporotic fractures, BMD, or bone turnover.

### Relationship between bone imaging and structure parameters and IL6

Our study showed no difference in fractures, BMD, or TBS across the different quartiles of IL6. Interleukin 6 is a multifunctional cytokine that plays a pivotal role in the regulation of immune responses and bone marrow hematopoiesis. It is also involved in the regulation of osteoclastogenesis and bone resorption in many pathophysiological conditions. It exerts its biological activity by binding to specific receptors; only a small fraction is free [19]. The IL-6 receptor consists of two subunits: the ligand-binding low-affinity component soluble receptor (sIL-6R) and a signal transducing receptor (gp130) [43].

The literature on IL-6 is inconsistent. In one study that included men and women, Ding et al. found that IL-6 was consistently associated with reduced BMD [12]. Sansoni et al. found, after multiple regression, that only sIL-6R, but not IL-6, was a significant determinant of BMD in postmenopausal women [43]. Cauley et al. [44] found a higher mean concentration of IL6 in men who had experienced a hip fracture relative to those without, but the same association was not apparent in women [19]. Interleukin 6 is the only cytokine that shows diurnal variation and reaches its lowest point in the morning [45]. Moreover, it has been reported that circulating IL-6 concentrations across a 24-h time period and a transient increase of IL-6 level during the sleep period can be attributed, at least in part, to effects of having an IV catheter and/or difficulties with blood drawing [46]. The difficulties with reproducible measurements of IL-6 could explain the discrepancies in the literature.

### Relationship between bone imaging and structure parameters and TNF- α

Our study revealed no difference in fractures, BMD or TBS in the different quartiles of TNF-α. TNF-α, a multifunctional cytokine mainly produced by activated macrophages, is one of the most potent osteoclastogenic cytokines produced in inflammation. TNF-α is responsible for stimulating osteoclastic bone resorption in vitro, as well as in vivo. TNF-α is associated with various cell signaling systems via 2 types of cell surface receptor, namely TNFR I (p55) and TNFR II (p75). In pathological circumstances, it has been shown that TNFR I promotes osteoclastogenesis, whereas TNFR II acts as an inhibitor [47].

The Health Aging and Body Composition Study, which included 2985 men and women > 70 years old, showed that the fracture incidence increases with the cytokine level. Moreover, subjects in the highest level of TNF-α had a 33% increased risk of fracture, independent of other risk factors [19]. However, no information on patients with inflammatory disease was given, and the results were not adjusted for BMI. The discrepancy with our results could be explained by the short half-life of TNF-α in the circulation. Increases in serum concentrations of TNF-α are usually transient, whereas elevations in serum TNF-α soluble receptors are more constant [19].

In the present study, the method of cytokine measurement was similar. Venous blood samples also were drawn in the fasting state, allowed to clot and then kept at − 80 °C before assessment and sent in dry ice to the laboratory. Our lower limit of detection for TNF-α was 0.2 pg/ml [24].

### Strengths and limitations

To our knowledge, this is currently the largest population-based sample in which the associations between bone parameters (prevalent and incident fractures, BMD at two different sites and TBS) and inflammatory cytokines (hs-CRP, IL-6, IL-1β and TNF-α) within the normal range have been assessed in postmenopausal women, excluding participants with a chronic inflammatory disease. However, we should also mention some limitations. First, the duration was only 5 years and we measured circulating cytokines in the serum, but the highest concentrations of cytokines may be found in the bone microenvironment [19]. Secondly, we did not adjust our results to physical activity or medication use. For example, Nonsteroidal anti-inflammatory drugs (NSAIDS) or betablockers have a well-known impact on bone and could affect inflammatory parameters [19], and statins could inhibit TNF-α production and release and, thereby, inhibit IL-6 and CRP production [6]. Thirdly, we measured IL-6 and not sIL-6R or gp130, which are more stable than IL-6. The same applies for TNF-α (we did not measure TNF sRI or TNF sRII) and IL-1β (we did not measure IL-1ra). Fourthly, we did not measure bone serum markers (such as β Crosslaps) or vitamin D levels in our study.

Our study gives important information about postmenopausal Caucasian women, but cannot be generalized to men or to other ethnicities. Finally, we hypothesize that BMI itself could explain the link between aging and bone imaging and structure parameters degradation. Aging is associated with changes in body composition, including a decrease in lean mass and an increase in total-body fat, particularly abdominal adipose tissue [6]. In octogenarians, investigators have found that plasma levels of TNF-α are correlated linearly with systemic levels of leptin, which is proportional to the amount of fat mass [6]. In our study, the oldest women had the highest BMI.

## Conclusions

In conclusion, in this large population-based cohort of postmenopausal women, we did not find a relation between so-called “inflammaging” and bone imaging and structure parameters, measured in terms of prevalent and incident fractures, lumbar spine and hip BMD or TBS. Future studies in this area should probably include body composition data.

## Data Availability

The data that support the findings of this study are available from the *Colaus Study* but restrictions apply to the availability of these data, which were used under license for the current study, and so are not publicly available. Data are however available from the authors upon reasonable request and with permission of the *Colaus Study*.

## Abbreviations

BMD: Bone mineral density
BMI: Bone mass index
COPD: Chronic obstructive pulmonary disease
DMARDS: Disease-modifying antirheumatic drugs
FRAX: Fracture Risk Assessment Tool
HRT: Hormone replacement therapy
hs-CRP: High-sensitivity C-reactive protein
IL-*1β*: Interleukin-*1β*
IL-6: Interleukin-6
NSAIDS: Nonsteroidal anti-inflammatory drugs
OA: Spinal osteoarthritis
OP: Osteoporosis
pQCT: Peripheral quantitative computed tomography
RA: Rheumatoid arthritis
RANK: Receptor activator of nuclear factor—*k*B
SLE: Systemic lupus erythematosus
TBS: Trabecular bone score
TNF-α: Tumor necrosis factor-α
VFA: Vertebral fracture assessment
